# 早期非小细胞肺癌立体定向放射治疗后的放射性肺炎

**DOI:** 10.3779/j.issn.1009-3419.2014.04.11

**Published:** 2014-04-20

**Authors:** 露 陈, 娅琴 赵, 峰 许

**Affiliations:** 610041 成都，四川大学华西医院肿瘤中心 Cancer Center, West China Hospital, Sichuan University, Chengdu 610041, China

**Keywords:** 立体定向放射治疗, 肺肿瘤, 放射性肺炎, Stereotactic body radiation therapy, Lung neoplasms, Radiation pneumonitis

## Abstract

随着放疗技术的进步，立体定向放射治疗（stereotactic body radiation therapy, SBRT）在早期非小细胞肺癌（non-small cell lung cancer, NSCLC）中得到了广泛应用，其不仅是不可手术的早期NSCLC的标准治疗方法，也是可手术的早期NSCLC的治疗方法之一。放射性肺炎（radiation pneumonitis, RP）是SBRT治疗后最常见的并发症。SBRT独特的分割计划和剂量分布使其不仅在治疗效果上和常规分割放疗不同，而且治疗后引起的RP和常规分割放疗引起的RP也有所不同。RP的发生可降低患者的生活质量，甚至导致治疗失败。因此，降低RP的风险对提高患者的生活质量和肿瘤的控制率有重要意义。本文就SBRT在早期NSCLC治疗中应用、治疗后RP的发生率、影像学表现以及预测因素方面作一综述。

## Abstract (en)

With the development of radiation technology, stereotactic body radiation therapy (SBRT) has been widely used in early stage non-small cell lung cancer (NSCLC). It is not only the standard therapy for medically inoperable early-stage NSCLC, but also one of the therapies for operable early-stage NSCLC. Radiation pneumonitis (RP) is one of the most common adverse effects after SBRT, it may reduce the patients' quality of life, even cause treatment failure. Therefore, in order to improve the patients' quality of life and enhance local control rate of tumor, it is important to reduce the risk of RP. The unique fractionation schemes and the dose distribution of SBRT make it not only different from conventional fraction radiation therapy in treatment outcomes, but also in the incidence of radiation pneumonitis. This article reviews the applying of SBRT for early stage NSCLC, the incidence of radiation pneumonitis, radiological appearance after SBRT and predictive factors.

自1995年Blomgren^[1]^首次报道立体定向放射治疗（stereotactic body radiation therapy, SBRT）用于颅外肿瘤治疗的结果以来，SBRT被越来越广泛的应用于颅外肿瘤的治疗。SBRT目前已成为不可手术的早期非小细胞肺癌（non-small cell lung cancer, NSCLC）的标准治疗方法^[2]^。有证据^[3]^显示对于那些存在高手术风险的患者来说，采用SBRT治疗能获得与手术相当的治疗效果。放射性肺炎（radiation pneumonitis, RP）是SBRT治疗早期NSCLC后最常见的并发症之一。研究^[4-9]^显示SBRT治疗后RP的发生率超过50%，其中有症状（≥2级）的RP为9%-28%。虽然报道中大多数的RP为2级，但因大多数不可手术的NSCLC患者合并有内科疾病，即使是2级的RP也可能危及患者生命。RP可能引起一些慢性并发症，如肺纤维化、肺功能不全等，降低患者的生活质量，导致治疗失败。因此，降低RP的风险对提高肿瘤的控制率和患者的生活质量有重要意义。SBRT独特的分割计划和剂量分布使其不仅在治疗效果上和常规分割放疗不同，治疗后引起的RP和常规分割放疗引起的RP也有所不同^[10]^。

## 1 SBRT在早期NSCLC中的应用

手术切除目前仍然是早期（T1-2N0M0）NSCLC的标准治疗方法，5年生存率为60%-70%，也有个别报道^[11]^显示5年生存率达到80%。但在临床中肺癌患者常因内科合并症，如慢性阻塞性肺疾病、心血管疾病、糖尿病等，而不能接受手术治疗。对于这些不能手术的患者，既往常采用常规分割放射治疗或者随访观察。但采取随访观察的患者中，有近50%的患者最终死于癌症^[12]^，而采用常规分割放疗的肿瘤控制率和生存率明显低于手术切除^[13-15]^。增加肿瘤的放疗剂量能提高肿瘤的局部控制率^[16]^，但肺部放疗的剂量常常受到肺、心脏、食道及脊髓等周围重要器官的限制。因此常规分割放疗无法通过提高的照射剂量来提高肿瘤的控制率。随着放疗技术的发展，SBRT弥补了这一缺陷。SBRT采用精确的定位系统，使高剂量集中在靶区，在提高肿瘤区域剂量的同时，极大程度地减少周围正常器官和组织的照射剂量^[17]^。美国放射治疗肿瘤协作组（Radiation Therapy Oncology Group, RTOG）开展的一项多中心临床研究RTOG-0236^[18]^，结果显示3年肿瘤局部控制率为97.6%，3年生存率为55.8%，不良反应主要包括缺氧、RP、肺功能减退，3级和4级不良反应的发生率为15.2%，无5级不良反应发生。Takeda等^[19]^对63例早期不可手术的NSCLC患者进行了回顾性分析，其中Ⅰa期（T1N0M0）患者38例，Ⅰb期（T2N0M0）患者25例，结果显示Ⅰa期、Ⅰb期患者的3年局部控制率和3年生存率分别为93% *vs* 96%（*P*=0.86）、90% *vs* 63%（*P*=0.09），4.7%的患者发生≥2级的RP。当生物有效剂量（biologically effective dose, BED）≥100 Gy时，3年局部控制率超过90%，3年生存率在43%-83%^[18, 20-24]^。虽然各个研究报道的局部控制率和生存率存在差别，但这些研究均证实SBRT治疗不可手术的早期NSCLC是安全、有效的。

SBRT除了用于早期不可手术的NSCLC患者，近年也有研究将SBRT运用于能手术治疗的早期NSCLC患者。Onishi等^[25]^对87例可手术的早期NSCLC患者进行了回顾性分析，这些患者因拒绝手术治疗，而选取了SBRT治疗，放疗剂量为45 Gy-72.5 Gy，分割次数为3次-10次，随访时间超过55个月，T1期、T2期的局控率分别为92%、73%，5年生存率分别为72%、62%，结果提示SBRT有可能达到手术治疗的效果。一项来自荷兰的研究^[26]^也报道了类似的结果。2004年，日本临床肿瘤协会（Japan Clinical Oncology Group, JCOG）开展了一项针对可手术治疗的Ⅰa期NSCLC患者的Ⅱ期临床研究（JCOG0403）^[27]^，研究中纳入65例患者，单次照射剂量为12 Gy，共4次。中位随访时间为45个月，3年生存率为76%，3年局部控制率为69%。治疗相关毒性包括1例胸痛、2例呼吸困难、1例缺氧、2例RP，没有患者发生4级以上不良反应。目前将SBRT用于可手术的患者的证据尚不充足，SBRT也不能如手术治疗一样，可以进行淋巴结切除以进一步明确分期^[28]^，这就限制了SBRT在可手术的早期NSCLC患者中的使用。目前有许多正在进行的针对SBRT用于可手术的早期NSCLC患者的临床研究，如RTOG0618、ACOSOG Z4099/RTOG1021、STARS、ROSEL等^[29]^。相信这些研究的结果能为我们提供更多的证据和指导。

## 2 RP的发生率

RP是SBRT治疗早期NSCLC后最常见的并发症之一。研究^[4-9]^显示SBRT后RP的发生率超过50%，其中有症状（≥2级）的RP为9%-28%。Takeda等^[30]^的研究纳入128例患者，共133个靶病灶，中位随访时间为12个月（5个月-45个月）。发生0级、1级、2级、3级的RP分别为27%、52%、16%、5%，未发现4级和5级的RP。Barriger等^[8]^的一项回顾性研究纳入251例患者，放疗剂量为24 Gy-72 Gy，分3次-5次照射，中位随访时间为17个月，治疗后42例（17%）患者出现RP，其中1级8%，2级7%，3级2%，4级0.4%，没有5级的RP发生。一项来自日本的大型多中心回顾性研究^[31]^纳入257例患者，单次分割剂量为4.4 Gy-35 Gy，分割次数为1次-14次，中位随访时间为38个月，其中28例（10.9%）患者发生有症状（> 1级）的RP，这些患者中有25例（89%）在治疗前合并有肺纤维化或肺气肿。14例患者出现2级（5.4%）RP，1例（0.4%）患者出现慢性节段性支气管炎、支气管管壁增厚，最终出现外周肺不张。其他不良反应包括食管炎（0.8%）、放射性皮肤炎（1.2%）、肋骨骨折（1.6%）。在Yamashita等^[4]^的一项研究中，0级-5级的放射性肺炎发生率分别为32%、40%、8%、4%、4%、12%。在Matsuo等^[32]^的研究纳入74例患者，结果中只记录了有症状的（≥2级）的RP，其中2级18.9%，3级1.4%，无4级和5级的放射性肺炎发生。由于各个研究采用的放疗计划、治疗技术、剂量参数以及RP分级标准的不同，各研究得到的结果差异也较大。虽然报道中很少有患者发生重度的RP，但因大多数不可手术的NSCLC患者为高龄患者且合并有内科疾病，即使是中度的RP也可能危及患者生命，所以在临床中应特别注意RP的防治。

## 3 SBRT治疗后的影像学表现

SBRT后的影像学表现可以分为早期（急性）反应和晚期（慢性）反应。早期反应，即RP，常发生在放疗后的6个月内；晚期反应，即放射性肺纤维化（radiation lung?brosis, RLF）发生在放疗后的6个月之后。在大多数情况下，正常肺组织的影像学改变发生在SBRT后的3个月之后，而RP的临床症状常发生在治疗后的3个月-6个月。在SBRT治疗后的6个月-9个月内RP逐渐向RLF转归，RLF也可以出现在那些没有发生RP的患者中^[33]^。RLF的影像学表现在SBRT治疗后的1年-2年内维持稳定，基本不发生变化^[33-36]^。

既往的研究对SBRT治疗后的急性期和慢性期CT表现进行了详细的描述^[35, 37]^。早期的CT表现主要和炎症相关，SBRT后的RP主要表现有弥漫性结节影；弥漫性磨玻璃样影；斑片状实变和磨玻璃样影；斑片状磨玻璃样阴影；无明显变化。晚期CT表现和纤维化相关，主要表现为：与常规放疗引起的RLF类似的变化，如实变、体积减小、支气管扩张，但程度较轻；团块样改变；瘢痕样改变；无明显变化。RP常从计划靶区（planning target volume, PTV）周围开始出现，最终在RLF出现前和PTV的区域重合在一起，但是致密实变区并不总是和PTV的范围一致，实变区的形状和位置会在第1年内呈动态变化^[38]^。Hof等^[39]^在单次分割的SBRT中发现，放疗剂量和正常组织改变的范围呈正相关，即放疗剂量越大，正常组织改变的范围越大。Palma等^[40]^的研究也得到了相同的结论。他们的研究表明CT的密度随着放疗剂量、PTV的范围、放疗时间的增加而增加。在接受放疗剂量 > 6 Gy区域密度开始增加，当放疗剂量 > 20 Gy时，密度明显增加，而在放疗剂量 > 40 Gy时，密度不再随放射剂量增加而增加。

近年来逐渐有研究者将PET/CT用于SBRT治疗肺癌后的疗效评价。在Henderson等^[41]^的研究中，他们使用PET/CT对不可手术的Ⅰ期NSCLC患者进行疗效评估。在SBRT前以及治疗后的2周、26周、52周分别行PET/CT检查，研究发现在SBRT治疗前SUV值低的患者在治疗的两周后SUV值可能升高，而治疗前SUV值高的患者，在治疗后的第2周后，SUV开始降低。在13例肺原发肿瘤的患者中，有6例患者在SBRT治疗后的12个月后PET/CT检查的SUV值> 3.5，但是在随后的随访中没有任何证据显示存在局部复发。一项来自日本的研究^[42]^显示在SBRT治疗的早期，特别是在治疗后的6个月内容易出现FDG摄取增高，随后逐渐降低；而SBRT治疗不久后出现FDG摄取中度升高并不能表明有肿瘤残余。也有研究表明PET/CT的表现和治疗反应存在良好的相关性。Coon等^[43]^的研究对51例采用SBRT治疗的患者进行研究，研究中包括肺原发NSCLC 26例、复发12例、肺转移瘤13例，在治疗后疗效评价为稳定、部分缓解、完全缓解、疾病进展的情况下，SUV值下降分别为28%、48%、94%和4%。Feigenberg等^[44]^的研究发现SBRT治疗NSCLC后3个月SUV值的降低和局部控制率相关，而有6%的患者在治疗后的9个月至1年内SUV值未降低出现了局部复发。Takeda等^[45]^的研究表明，最大标准摄入值SUVmax是局部复发重要的预测因素，当SUVmax较大时可考虑增加放射剂量以提高局部控制率。Horne等^[46]^的研究也得到了类似的结果，该研究指出SUVmax可作为无进展生存期（progression-free survival, PFS）的预测指标。

目前对于PET/CT用于SBRT治疗后的随访是否优于CT仍然存在争议，但根据文献报道来看，CT用于SBRT治疗早期NSCLC后的随访和疗效评价仍占着主导地位^[47]^。

## 4 预测因素

在肺的常规分割放疗中，剂量学参数可以作为RP的预测因子，限制照射剂量能减少RP的风险。在NSCLC的根治性常规分割放疗中，肺V20≤30%-35%，肺平均剂量（mean lung dose, MLD）≤20 Gy-23 Gy，能使RP发生风险≤20%^[48]^。而目前能用于预测SBRT治疗早期NSCLC引起RP的剂量学参数尚无统一标准。Barriger等^[8]^发现双肺总的MLD和V20与RP相关，研究纳入251例患者，其中23例（9.2%）发生2级-4级RP，这些患者照射总剂量为42 Gy-60 Gy，每次的照射8 Gy-28 Gy，当MLD≤4 Gy时，4.3%的患者发生2级-4级RP，而当MLD > 4 Gy时，17.6%的患者发生2级-4级RP（*P*=0.02）。Chang等^[49]^的一项回顾性研究显示同侧肺MLD是RP的重要预测指标，当MLD≥9.1 Gy时，RP的发生率为93%，当MLD < 9.1 Gy时，RP的发生率为7%。最近的一项前瞻性研究^[50]^表明对侧肺MLD和内靶区（internal target volume, ITV）的大小是3级以上RP的预测指标。该研究中的回归分割分析（recursive partitioning analysis, RPA）显示当对侧肺MLD≥3.6 Gy时，为RP高危组，发生RP的风险为37.5%；对侧肺MLD < 3.6 Gy且ITV≥145 cm^3^时，为中危组，发生RP的风险为28.6%；对侧肺MLD < 3.6 Gy且ITV < 145 cm^3^时，为低危组，发生RP的风险为1.9%。此外，大于或等于某特定剂量的肺组织相对体积如V5、V10、V20等、PTV、肿瘤大小也与RP相关。Ong等^[7]^对肺肿瘤体积 > 80 cm^3^的18例患者进行的研究发现，对侧肺V5、双肺总的V10和V5、MLD、PTV与RP相关，其中相关性最好的指标为对侧肺V5，将对侧肺V5限制在26%以内可以减少RP的发生。Guckenberger等^[6]^对59例患者的回顾性分析显示MLD、同侧肺V2.5-V50和RP的发生相关。Matsuo等^[32]^的研究纳入74例NSCLC患者，研究中2级及以上的RP为20.3%，肺V25和PTV为RP重要的预测指标，当PTV≥37.7 mL，肺V25≥4.2%时，发生2级及以上的RP风险为50%。最近一项来自加拿大的前瞻性研究^[51]^对185例早期不可手术的NSCLC患者进行了分析，结果显示T2（> 3 cm）期患者发生≥2级的RP的概率高于T1（< 3 cm Ⅱ期患者（17.9% *vs* 4.4%, *P*=0.02），表明肿瘤的大小和RP的发生存在相关性。

除剂量学参数外，一些其他的因素也可能影响SBRT治疗后RP的发生。有研究^[52]^指出Ⅱ型肺泡表明抗原（Krebs von den Lungen-6, KL-6）也是RP的一个预测指标。KL-6是一种肺腺癌相关抗原，在Ⅱ型肺泡细胞和细支气管上皮细胞中高表达。Hara等^[53]^对16例接受单次SBRT治疗患者的血清KL-6水平进行了检测，结果显示SBRT治疗结束后2个月的血清KL-6较治疗前增加的相对值和3级RP的发生密切相关。Iwata等^[54]^对160例接受SBRT治疗的患者的血清KL-6水平进行了分析，SBRT治疗前的血清KL-6在单因素和多因素分析中均有统计学意义，并指出在SBRT治疗中应该对治疗血清KL-6≥300 U/mL的患者进行密切随访以防RP的发生。Yamashita等^[55]^对重度（4级和5级）RP的危险因素进行了研究，研究中对117例患者进行了回顾性分析，其中肺原发肿瘤为74例，转移或复发为43例，有9例（7.7%）患者发生重度RP，结果显示治疗前的血清KL-6、肺表面活性蛋白（surfactant proteins D, SP-D）、治疗前间质性肺炎的CT影像和重度RP的发生相关，建议SBRT治疗前对患者进行胸部CT检查，明确是否存在间质性肺炎，以及血清KL-6和血清SP-D水平的检测。研究中通过这三项指标的初筛，重度RP的发生率由18.8%降至3.5%。Takeda等^[30]^的研究显示RP在胸部平片的影像出现的越早，出现重度RP的可能越大。RP的影像学表现出现在治疗后的最初2个月内，发生3级RP的风险为40%，而RP的影像学表现出现在治疗后的3个月之后，发生RP的风险为1.2%。

## 5 结语

综上所述，SBRT不仅在不可手术的早期NSCLC患者中取得了良好的局部控制率和生存率，而且在可手术的早期NSCLC患者中也有一定的应用价值。目前关于SBRT治疗后的RP的剂量-体积参数方面的研究仍十分有限，尚无公认的剂量学参数可以预测RP。因此，未来需要更多大样本的前瞻性研究提供数据支持，以便更好的指导临床。
